# Exploration of new space elicits phosphorylation of GluA1(Ser831) and S6K and expression of Arc in the hippocampus in vivo as in long-term potentiation

**DOI:** 10.1186/s13041-024-01100-x

**Published:** 2024-06-10

**Authors:** Roberta Cagnetta, Jean-Claude Lacaille, Nahum Sonenberg

**Affiliations:** 1 Department of Cell Biology and Program in Neuroscience, Harvard Medical School, Boston, MA 02115 USA; 2 Department of Biochemistry and Goodman Cancer Institute, McGill University, Montreal, QC H3A 1A3 Canada; 3 Department of Neurosciences, Center for Interdisciplinary Research On Brain and Learning, Research Group On Neural Signaling and Circuitry, University of Montreal, Montreal, QC H3T1J4 Canada

**Keywords:** Synaptic plasticity, LTP, LTD, Behaviour, Exploration of new space, Non-aversive behavioural task, AMPA receptor code, p-GluA1(Ser831), p-GluA1(Ser845), mTORC1-S6K signalling pathway, Arc

## Abstract

The brain responds to experience through modulation of synaptic transmission, that is synaptic plasticity. An increase in the strength of synaptic transmission is manifested as long-term potentiation (LTP), while a decrease in the strength of synaptic transmission is expressed as long-term depression (LTD). Most of the studies of synaptic plasticity have been carried out by induction via electrophysiological stimulation. It is largely unknown in which behavioural tasks such synaptic plasticity occurs. Moreover, some stimuli can induce both LTP and LTD, thus making it difficult to separately study the different forms of synaptic plasticity. Two studies have shown that an aversive memory task – inhibitory avoidance learning and contextual fear conditioning – physiologically and selectively induce LTP and an LTP-like molecular change, respectively, in the hippocampus in vivo. Here, we show that a non-aversive behavioural task – exploration of new space – physiologically and selectively elicits a biochemical change in the hippocampus that is a hallmark of LTP. Specifically, we found that exploration of new space induces an increase in the phosphorylation of GluA1(Ser831), without affecting the phosphorylation of GluA1(Ser845), which are biomarkers of early-LTP and not NMDAR-mediated LTD. We also show that exploration of new space engenders the phosphorylation of the translational regulator S6K and the expression of Arc, which are features of electrophysiologically-induced late-LTP in the hippocampus. Therefore, our results show that exploration of new space is a novel non-aversive behavioural paradigm that elicits molecular changes in vivo that are analogous to those occurring during early- and late-LTP, but not during NMDAR-mediated LTD.

## Introduction

The brain adapts to experiences by alteration of synaptic transmission, that is synaptic plasticity [1]. Synaptic plasticity is bidirectionally modifiable. An increase in the strength of synaptic transmission is exhibited as long-term potentiation (LTP). A decrease in the strength of synaptic transmission is manifested as long-term depression (LTD) [1]. LTP consists of two phases: early and late. The early-LTP is protein synthesis-independent, whereas the late-LTP (i.e. when the LTP is maintained for more than 1 h after its induction) is protein synthesis-dependent [1]. LTD is protein synthesis-independent when it is mediated by NMDAR but protein synthesis-dependent when it is mediated by mGluR [1, 2].

An ‘AMPA receptor code’ has been proposed to predict and distinguish between the different forms of synaptic plasticity [3, 4]. Namely, early-LTP elicits the phosphorylation of Ser831 of the GluA1 subunit of the AMPA receptor, without affecting the phosphorylation of Ser845, in the hippocampus [5, 6]. In contrast, NMDAR-mediated LTD elicits the dephosphorylation of Ser845 of the GluA1 subunit of the AMPA receptor, without affecting the phosphorylation of Ser831, in the hippocampus [5, 7].

Studies of synaptic plasticity routinely use electrophysiological stimulation for induction (e.g. high frequency stimulation to induce LTP, low frequency stimulation to induce LTD). However, the behavioural relevance of such induction paradigms is largely unknown. Moreover, some stimuli can elicit both LTP and LTD [8], thus making it difficult to parse their respective mechanisms of synaptic plasticity. Importantly, two aversive memory tasks – inhibitory avoidance learning and contextual fear conditioning – were found to physiologically and selectively induce LTP and an LTP-like molecular change (i.e. phosphorylation of GluA1(Ser831) and unchanged p-GluA1(Ser845)), respectively, in the hippocampus [6, 9].

In the present study, we show by biochemical approaches, referring to the ‘AMPA receptor code’ and hallmarks of protein synthesis-dependent synaptic plasticity, that a non-aversive behavioural task – exploration of new space – physiologically and selectively elicits LTP-like, but not NMDAR-mediated LTD-like, molecular changes in the hippocampus in vivo. Specifically, we found that exploration of new space induces: (i) phosphorylation of GluA1(Ser831) without affecting p-GluA1(Ser845), (ii) an increase in the phosphorylation of the translational regulator S6K, and (iii) an increase in the expression of Arc protein. Thus, exploration of new space is a novel non-aversive behavioural paradigm that elicits LTP-like biochemical changes in the hippocampus in vivo.

## Materials and methods

### Animals

Wild-type mice were from the Jackson Lab. Animals were group housed with 3–5 adult males per home cage (19.1 cm × 29.2 cm × 12.7 cm) and maintained on a 12 h light–dark cycle (lights on at 7 am). Food and water were available ad libitum at all times.

### Ethics

All procedures were approved by the McGill Animal Care Committee and complied with the Canadian Council for Animal Care guidelines.

### Behavioural task

Male mice used for behavioural testing were 8–14 weeks old. Mice were housed in the facility for a minimum of 1 week prior to behavioural experiments. Mice of both the control and experimental conditions were adjusted to the testing room for at least 30 min and handled for 1 min each in the testing room for 3 consecutive days before the experiments. Mice of both the control and experimental conditions were adjusted to the testing room for at least 30 min before the start of experiment. The behavioural experiments were performed between the hours of 7 am – 2 pm. The apparatus for the exploration of new space behavioural task consisted of an empty white square wooden box (48 cm × 48 cm × 48 cm) in which the mouse was allowed to roam freely for 15 min. A camera was mounted above the box for recording. The mouse was placed in the middle of the box at the start of the test. The boxes were cleaned between each mouse with an odour-less disinfectant. As control, mice were brought to the testing room and kept in their familiar space, that is their cage, on the day of the experiment.

### Western blotting

Probing for biochemical hallmarks of synaptic plasticity was carried out on whole hippocampal lysates by western blotting. Bradford Protein Assay kit and spectrophotometry were used to determine the protein concentration. Bovine serum albumin was used as a standard curve for protein concentration and for normalizing amounts among samples. Proteins were separated on a 10% polyacrylamide gel. Proteins were transferred to a nitrocellulose membrane, which was incubated with the antibody of interest at 4 °C overnight in 5% BSA solution. The antibodies used were: anti-GluA1 (Abcam, ab183797), anti-p-GluA1(Ser845) (Thermo Fisher Scientific, 36–8300), anti-p-GluA1(Ser831) (Abcam, ab109464), anti-S6K1 (Cell signaling, 9202), anti-p-S6K(Thr389) (Cell signaling, 9205), anti-Arc (Santa Cruz Biotechnology, sc-17839), anti-α-tubulin (Santa Cruz Biotechnology, sc-23948). The blots were then incubated with HRP-conjugated secondary antibodies at room temperature for 1 h, followed by ECL-based detection.

### Statistics

Data were analyzed with PRISM 10 (GraphPad). Data are presented as mean and error bars represent s.e.m. Experiments were performed in at least two independent biological replicates. Details of statistical tests are presented in the figure legends.

*p < 0.05, **p < 0.01, ***p < 0.001, ns: non-significant.

## Results

### Exploration of new space elicits a rapid and transient increase in the phosphorylation of GluA1(Ser831) without affecting p-GluA1(Ser845) in the hippocampus 

**Fig. 1:**
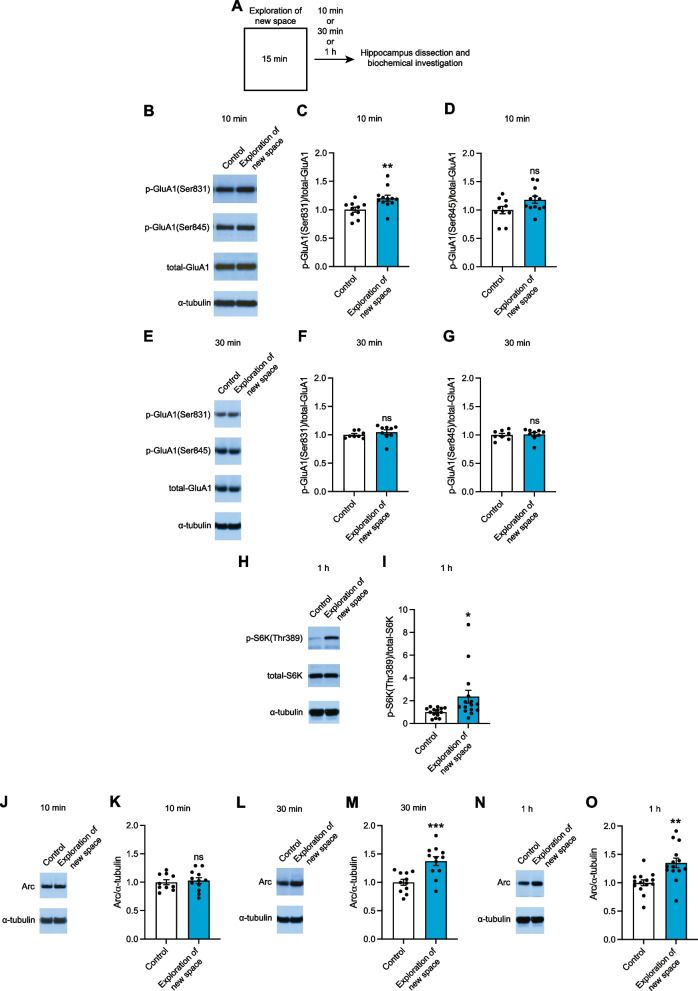
Exploration of new space induces LTP-like, but not NMDAR-mediated LTD-like, molecular changes in the hippocampus in vivo.** A** Schematic showing the experimental design. **B**, **C** and **D** Representative immunoblot (**B**) and quantification of p-GluA1(Ser831)/total-GluA1 (**C**) and p-GluA1(Ser845)/total-GluA1 (**D**) following 15 min exploration of new space in the hippocampus in vivo at a 10 min timepoint (unpaired t-test). **E**, **F** and **G** Representative immunoblot (**E**) and quantification of p-GluA1(Ser831)/total-GluA1 (**F**) and p-GluA1(Ser845)/total-GluA1 (**G**) following 15 min exploration of new space in the hippocampus in vivo at a 30 min timepoint (unpaired t-test). **H** and **I** Representative immunoblot (**H**) and quantification (**I**) of p-S6K(Thr389)/total-S6K following 15 min exploration of new space in the hippocampus in vivo at a 1 h timepoint (unpaired t-test). **J** and **K** Representative immunoblot (**J**) and quantification (**K**) of Arc following 15 min exploration of new space in the hippocampus in vivo at a 10 min timepoint (unpaired t-test). **L** and **M** Representative immunoblot (**L**) and quantification (**M**) of Arc following 15 min exploration of new space in the hippocampus in vivo at a 30 min timepoint (unpaired t-test). **N** and **O** Representative immunoblot (**N**) and quantification (**O**) of Arc following 15 min exploration of new space in the hippocampus in vivo at a 1 h timepoint (unpaired t-test)

We sought a behavioural paradigm to physiologically and selectively induce biochemical changes in vivo like those occurring during a form of synaptic plasticity. As a behavioural task mice explored new space for 15 min. 10 min after the exploration task, the hippocampus, which is required for encoding spatial information [10], was dissected to probe for biomarkers of LTP and LTD (Fig. 1A). We based our biochemical tests on the ‘AMPA receptor code’ that distinguishes between early-LTP when GluA1(Ser831) is phosphorylated whereas p-GluA1(Ser845) is unaffected, versus NMDAR-mediated LTD when GluA1(Ser845) is dephosphorylated whereas p-GluA1(Ser831) is unaffected [3–7]. At the 10 min timepoint, western blotting showed that exploration of new space elicits a 20% increase in the phosphorylation of GluA1(Ser831) (Fig. 1B and C), whereas p-GluA1(Ser845) does not change (Fig. 1B and D). This pattern, its timing and its magnitude are reminiscent of those observed in LTP induced ex vivo in response to theta burst stimulation and in vivo following inhibitory avoidance learning [5, 6].

Next, we measured p-GluA1(Ser831) and p-GluA1(Ser845) at a 30 min timepoint following exploration of new space (Fig. 1A). Western blot showed that p-GluA1(Ser831) returns to basal level within 30 min (Fig. 1E and F) and that p-GluA1(Ser845) remains unchanged (Fig. 1E and G). In agreement with previous results for inhibitory avoidance learning-induced LTP in vivo [6], these data show that the phosphorylation of GluA1(Ser831) is rapid and transient, rendering it a biomarker of early-LTP. Collectively, the data indicate that exploration of new space physiologically and selectively elicits biochemical changes in the AMPA receptor in the hippocampus in vivo like those occurring during early-LTP, but not NMDAR-mediated LTD.

### Exploration of new space increases p-S6K levels in the hippocampus 

Next, we probed for a feature of LTP to corroborate our working model concerning the ‘AMPA receptor code’. We examined the translational control signalling taking place in late-LTP, which is protein synthesis-dependent [1]. Previous work demonstrated that the translational regulator downstream of mTORC1, S6K, is phosphorylated in high frequency stimulation-induced late-LTP in the hippocampus [11, 12]. Therefore, we investigated the phosphorylation of S6K in the hippocampus using an anti-p-S6K(Thr389) antibody following exploration of new space at a 1 h timepoint (Fig. 1A), when late-LTP occurs [1]. In accord with the earlier findings [11, 12], western blotting revealed that the phosphorylation of S6K increases by 136% 1 h after exploration of new space (Fig. 1H and I). Thus, exploration of new space activates in the hippocampus a translational control mechanism which functions during late-LTP, that is the mTORC1-S6K signalling pathway.

### Exploration of new space induces an increase in Arc expression in the hippocampus 

Next, we probed for another important feature of late-LTP, which is the increase in the expression of the immediate early gene Arc via the ERK-MNK signalling pathway but not mTORC1 [12, 13]. Western blotting showed no increase in Arc at a 10 min timepoint following exploration of new space (Fig. 1J and K), but an increase of 37% at 30 min (Fig. 1L and M). No additional increase was detected at a 1 h timepoint (Fig. 1N and O). Thus, exploration of new space elicits an increase in the expression of Arc in the hippocampus as in late-LTP.

## Discussion

Our results reveal that exploration of new space physiologically and selectively elicits LTP-like, but not NMDAR-mediated LTD-like, molecular changes in the hippocampus in vivo. Our data confirm and extend previous work on inhibitory avoidance learning and contextual fear conditioning [6, 9], by showing that biochemical changes accompanying LTP occur in vivo following exploration of new space, which also involves memory but is a non-aversive behavioural task.

The AMPA receptor code is an optimal molecular marker to identify and distinguish LTP from LTD. Future studies will be needed to identify further biomarkers of the different forms of synaptic plasticity, for example by performing a comparative genome-wide investigation in LTP versus LTD. It should be noted that Arc is a feature but not a marker of late-LTP, as it is translated in late-LTP but also in mGluR-LTD and in response to neural activity [12–15]. The phosphorylation of S6K(Thr389) is also increased both in late-LTP and in mGluR-LTD [11, 12, 16]. Thus, we cannot rule out the possibility that mGluR-LTD may contribute to the exploration of new space-induced increase in p-S6K and Arc. Nevertheless, the exploration of new space-induced phosphorylation of S6K and expression of Arc, taken together with the phosphorylation of GluA1(Ser831) and unchanged p-GluA1(Ser845), bolster the findings that LTP-like molecular changes occur in the hippocampus following exploration of new space.

Our biochemical investigation was carried out in whole hippocampal lysates and the previous studies were similarly performed in the dorsal hippocampus and whole hippocampus [6, 9]. It would be important in future work to examine in which subregions of the hippocampus the phosphorylation of GluA1(Ser831) takes place following exploration of new space and other behavioural tasks. Indeed, the hippocampus exhibits specific associations with different behavioral patterns across its subregions – CA1, CA2, CA3 and dentate gyrus (e.g. [17–19]).

Our work warrants future electrophysiological studies to investigate whether exploration of new space is a novel behavioural paradigm to physiologically and selectively elicit LTP but not LTD in vivo. Studies will also be required to elucidate in which cognitive tasks LTD is manifested in vivo.

## Data Availability

Data and materials are available from the corresponding author upon request.

## Abbreviations

AMPA receptor: α-Amino-3-hydroxy-5-methyl-4-isoxazoleproprionic acid receptor
ERK: Extracellular signal-regulated kinase
GluA1: Glutamate receptor AMPA type subunit 1
LTP: Long-term potentiation
LTD: Long-term depression
mGluR: Group I metabotropic glutamate receptor
MNK: Mitogen-activated protein kinase-interacting serine/threonine-protein kinase
mTOR: Mechanistic/mammalian target of rapamycin
mTORC1: mTOR complex 1
NMDAR: N-methyl-D-aspartate receptor
S6K: Ribosomal protein S6 kinase
